# Supplementary material for: Definition and Quantification of Three-Dimensional Imaging Targets to Phenotype Pre-Eclampsia Subtypes: An Exploratory Study
Source: Int J Mol Sci. 2023 Feb 7;24(4):3240. doi: 10.3390/ijms24043240 (PMC9959375; doi:10.3390/ijms24043240)
Supplement: Supplementary file 1 [file ijms-24-03240-s001.zip › supplementary methods and figures v2.docx]

Definition and quantification of three-dimensional imaging targets to phenotype pre-eclampsia subtypes: an exploratory study

Sammy Hermans^1^, Jacob Pilon^1^, Dennis Eschweiler^2^, Johannes Stegmaier^2^, Carmen A. H. Severens – Rijvers^3^, Salwan Al-Nasiry^4^, Marc van Zandvoort^5,6^, Dimitrios Kapsokalyvas^1,7^

^1^Department of Genetics and Cell Biology, Maastricht University, Maastricht, The Netherlands.
^2^Institute of Imaging and Computer Vision, RWTH Aachen University, Aachen, Germany
^3^Pathology, Maastricht University Medical Centre, Maastricht (MUMC), The Netherlands.
^4^Obstetrics and Gynaecology, GROW, Maastricht University Medical Centre (MUMC), Maastricht, The Netherlands.

^5^Department of Genetics and Cell biology, GROW, CARIM, MHeNS, Maastricht University, Maastricht, The Netherlands.

^6^Institute for Molecular Cardiovascular Research IMCAR, University Hospital RWTH Aachen, Aachen, Germany

^7^Interdisciplinary Centre for Clinical Research IZKF, University Hospital RWTH Aachen, Aachen, Germany

**Supplementary methods**

***Vascular network extraction and quantification with VMTK***

A connected vascular segment was extracted with the “edit segment module”. Inside this module the *island* option was used to select the *largest connected segment*. From this segment a surface model (.vtk) was generated with the surface toolbox module. The surface model was loaded as input model into the VMTK centreline extraction module. Endpoints were manually selected. Eventually the generated network was imported to the centreline metrics module. This provided extensive diameter measurements along the network path.

Quantitative network output consisted of start- and end-point coordinates for each vessel segment, segment length, diameters, and tortuosity. The total network length was defined as the sum of the length of all individual segments. The amount of branchpoints were defined as the amount of unique start point coordinates. Branchpoint density was calculated by dividing the amount of branchpoints by total network length. Vessel segment partitioning was calculated by dividing the number of segments by the length of the network. Branchpoint morphology was assessed by evaluating identical start point coordinates.

***Nuclear segmentation***

Training data for Stardist were manually labelled in Labkit, with override option ‘on’. The example Jupyter Notebooks provided on <https://github.com/stardist/stardist/tree/master/examples/3D> were used to extract the code lines. Code lines were executed inside Visual Studio Code using the Jupyter Notebook extension. The interpreter language was set to python 3.7.9. First, the training data was assessed for suitability to be reconstructed with star-convex polygons. Based on the obtained reconstruction score, the model was trained with 96 rays, and taking anisotropy into account. Data augmentation was applied during training, and patch size was set to (16,128,128). Epoch was set to 1000 and the best training cycle was saved and assigned as the final model (weights.best). Images were normalized individually by img = normalize(X[num_img], 1,99.8, axis=axis_norm. axis_norm was set to (0,1,2)). For prediction, n_tiles was automatically calculated for each image individually to divide the image into patches of roughly size ~ (128,128,128).

***Setting probability threshold***The optimized threshold for prediction was automatically determined to be {"prob": 0.7575142354850506, "nms": 0.3}

***KnotMiner***

Knots were identified as protruding structures with at least 5 clustered cells. KnotMiner selected knot candidates based on intensity and weighted density threshold. Weighted density threshold (compared to density threshold) improved knot detection in cases where segmentation of individual nuclei in knots was imperfect. Resulting candidates were subsequently clustered with DBSCAN (1). Weighted density threshold and Minpoints were set to 5. Intensity threshold and epsilon had to be adjusted for each image separately (see below). Dominant presence of small knots required lower epsilon values than dominant presence of larger and elongated knots. Manual adjustments (e.g. removing selected non-trophoblast cells, or including non-selected obvious knots) were performed to refine the selections. Bridges and sprouts were manually identified based on 3D information and excluded from quantitative analysis.

**Manually adjusted parameters:**

| **File** | **Intensity threshold** | **Epsilon** | **Normalized** |
| --- | --- | --- | --- |
| EO-PE (30w,1d) Series 16 | 179 | 15 | 0.003 |
| EO-PE (30w,1d) Series 8 | 152 | 12 | 0.003 |
| EO-PE (30w,1d) Series 19 | 203 | 12 | 0.003 |
| EO-PE (30w,1d) Series 22 | 151 | 14 | 0.003 |
| Term (40w,4d) series 70 | 191 | 20 | 0.003 |
| Term (40w,2d) series 3 | 162 | 12 | 0.003 |
| Term (40w,4d) series 62 | 200 | 11 | 0.003 |
| LO-PE (37w,5d) series 12 | 172 | 18 | 0.005 |
| Petrem (31w,5d) | 206 | 11 | 0.003 |
| Petrem series 9 (32w,5d) | 198 | 15 | 0.003 |
| Petrem series 28 (32w,5d) | 75 | 15 | 0.003 |
| IUGR (40w,0d) series 9 | 157 | 10 | 0.003 |
| IUGR (40w,0d) series 2 | 112 | 13 | 0.003 |

**Table S1: List of images used in the graphs of the figures in the main text.**

| **Figure** | **Images used:**  **Case (** **Sample #: : number of images)** |
| --- | --- |
| Figure 2I | EO-PE (4:4, 5:1),  LO-PE (6:2),  Preterm control (8:3, 9:1)  Term control (1:1, 2:4, 3:1)  IUGR (7:2) |
| Figure 2J and 2K | EO-PE (4:4),  LO-PE (6:1),  Preterm control (8:2, 9:1)  Term control (1:1, 2:2)  IUGR (7:2) |
| Figure 3E | Preeclapmsia (4:7, 6:5) |
| Figure 3F | Term control (1:1, 2:7, 3:2) |
| Figure 3G | EO-PE (4:7),  LO-PE (6:5),  Term control (1:4, 2:4, 3:4)  Preterm control (8:3, 9:2)  IUGR (7:3) |
| Figure 4C | EO-PE (4:8),  LO-PE (6:5),  Preterm control (8:3)  Term control (1:2, 2:5, 3:2)  Term IUGR (7:3)  IUGR hypertension (10:1)  2^nd^ trimester placenta (11:2) |
| Figure 4D and 4E | EO-PE (4:3),  LO-PE (6:4),  Preterm control (8:2)  Term control (2:3)  Term IUGR (7:2) |


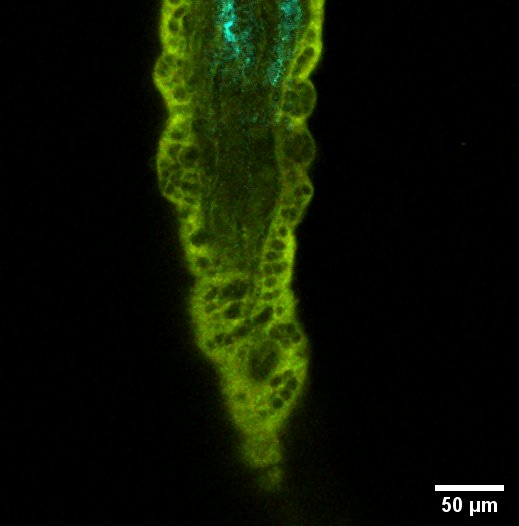

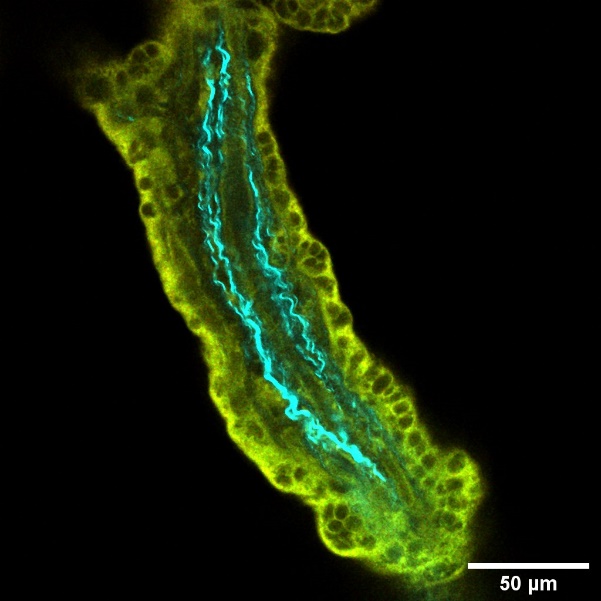


**Spontaneous idiopathic pre-term birth**


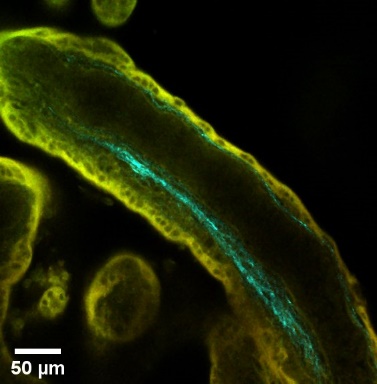


**EO-PE**


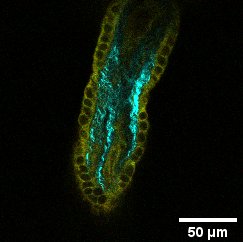


**Term control**

**Gestational hypertension & IUGR**


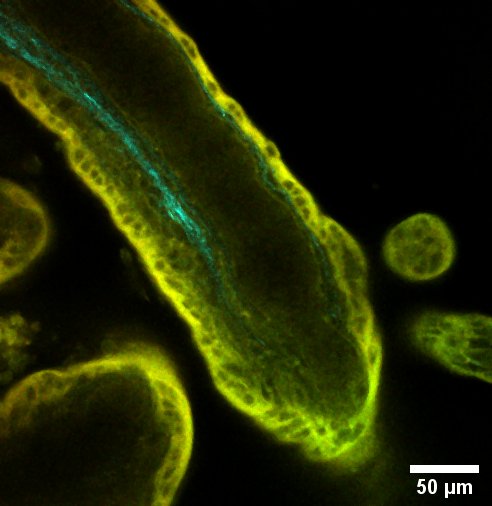


B

A

C

D

**Figure S1: Trophoblast structure in villi placenta with varying clinical images.** Placenta from different clinical pictures was imaged without fluorescent labelling to assess trophoblast structure. A: Spontaneous preterm birth placenta. Similar to B: EO-PE placenta, the trophoblast layer of preterm placenta (A) had disorganized appearance, but prominent presence of knots was rare. Although gestational age of pre-term placenta is better matched with gestational age of the investigated EO-PE placenta, placental abnormalities for pre-term labor cannot be ruled out. C: term control placenta illustrating the organized, single layered, trophoblastic arrangement. D: Placenta from pregnancy with gestational hypertension and growth restriction (ARED*) illustrates significant knotting. Green: villous tissue/ trophoblast, cyan: collagen. Scale bar: 50µm

*ARED=absent or reversed end diastolic flow, a clinical observation on doppler ultrasound which is potentially relevant for placental phenotyping/classification (5).

**EO-PE Term control**


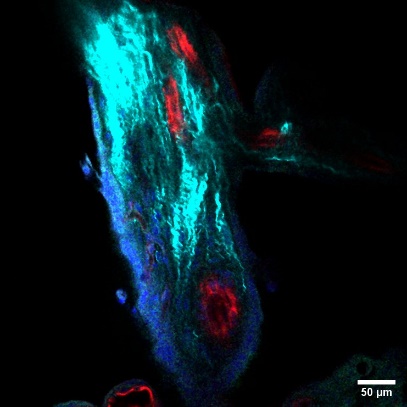

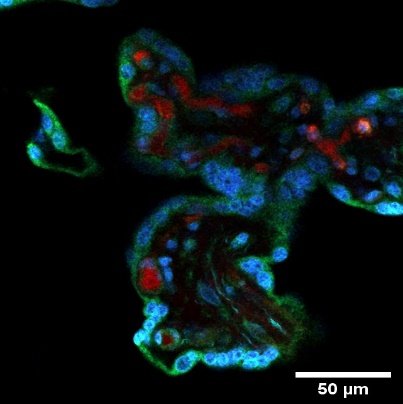

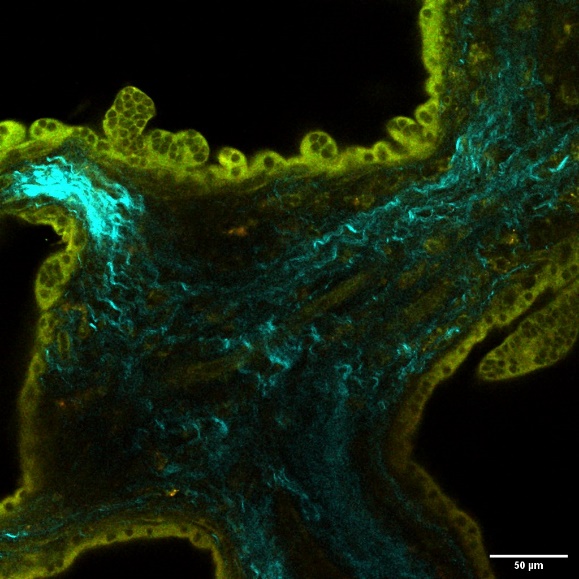

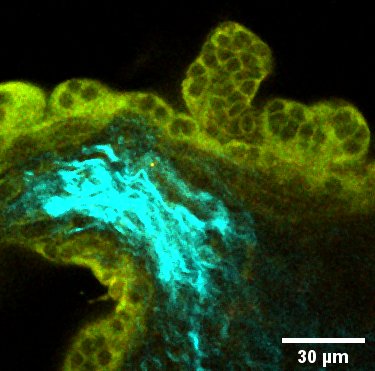

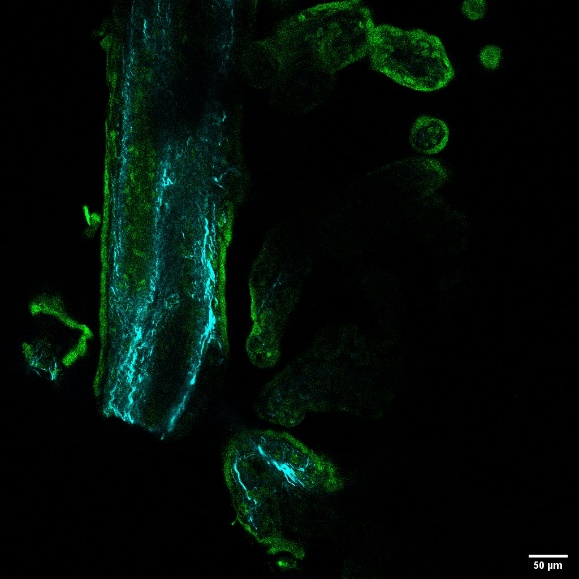


A

B

C

D

**
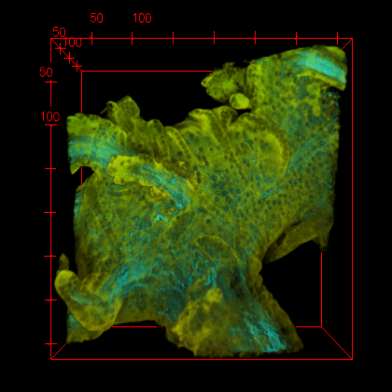
**


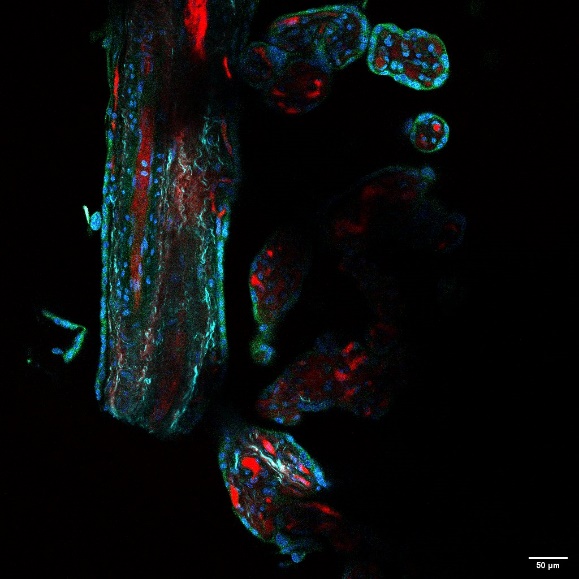


**Figure S2: Imaging targets fibrosis and vasculosyncytial membranes are visualized by the developed imaging protocol.** A: stem villous of EO-PE placenta without additional labelling. Besides prominent syncytial knotting, a fibrotic region is illustrated. B: EO-PE stem villous with central arteries and high collagen signal. Fibrotic regions were mostly observed in larger villi (stem villi). C: Terminal villi of term control placenta with little to no collagen signal. Vasculosyncytial membranes are illustrated as regions of cytoplasm void of nuclei, with a vessel closely aligned (blue arrows). D: The green arrow points to a stem villous of term control placenta. Collagen signal is mainly present in the stem villous and limited to absent in smaller villi. Green: villous tissue/ trophoblast, red: vessel, blue: nuclei, cyan: collagen. Scale bar: 50µm.

B

A


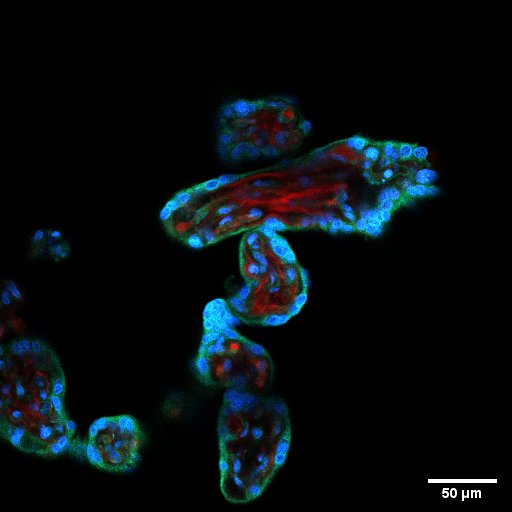

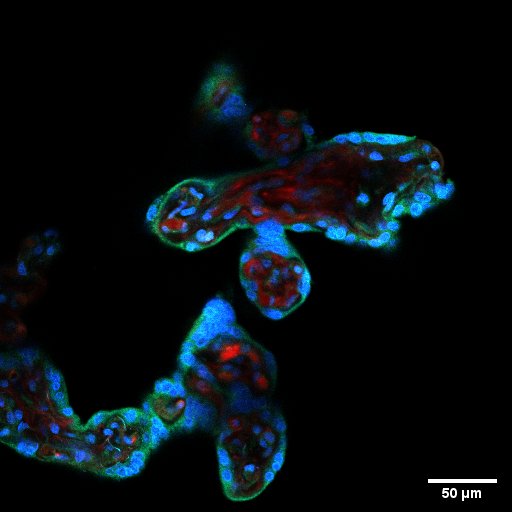

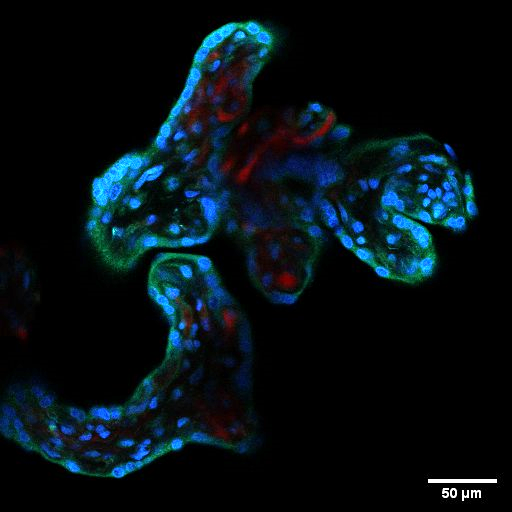

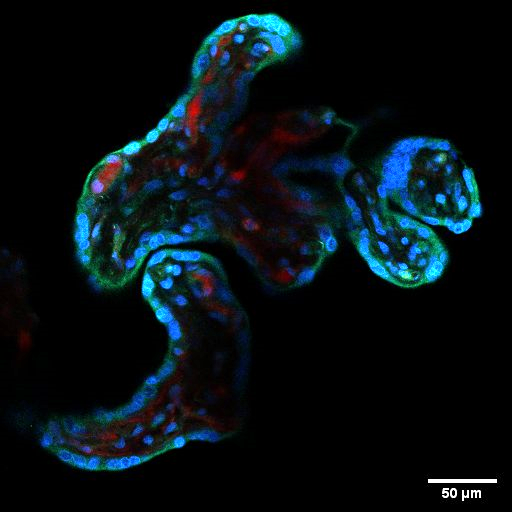


D

C

**Figure S3: Relevance of 3D information for bridge identification and the relation of knots with vasculosyncytial membranes.** Term placenta was imaged according to the developed protocol. A: The red arrow on a 2D slice appears to point to a syncytial knot. This example illustrates misinterpretation without additional 3D information. The white arrow points to syncytial bridge that is already identified as such on this 2D slice. B: On a 2D slice deeper in the same 3D image stack, the nuclear accumulation from A is clearly identified as a syncytial bridge (white arrow). C: The red arrow points to a ‘knot’ which appears to be formed to create a vasculo-syncytial membranes. Vasculosyncytial membranes are localised regions of the villous membrane void of nuclei or stroma and close to fetal blood vessels, resulting in a very thin barrier separating the maternal and fetal circulations. These thin single layers of syncytiotrophoblast in close contact with placental vasculature represent the major areas of feto-maternal exchange (6). D: Increasing z-size with two steps (10µm) in comparison to image C, illustrates a VSM right below the knot. Blue arrows point to VSMs. Scale bar: 50µm


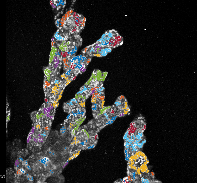

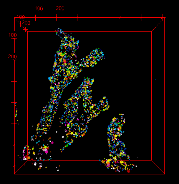
**
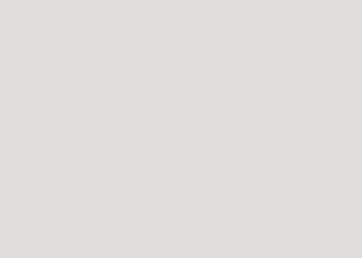
**

**KnotMiner**

**Stardist**


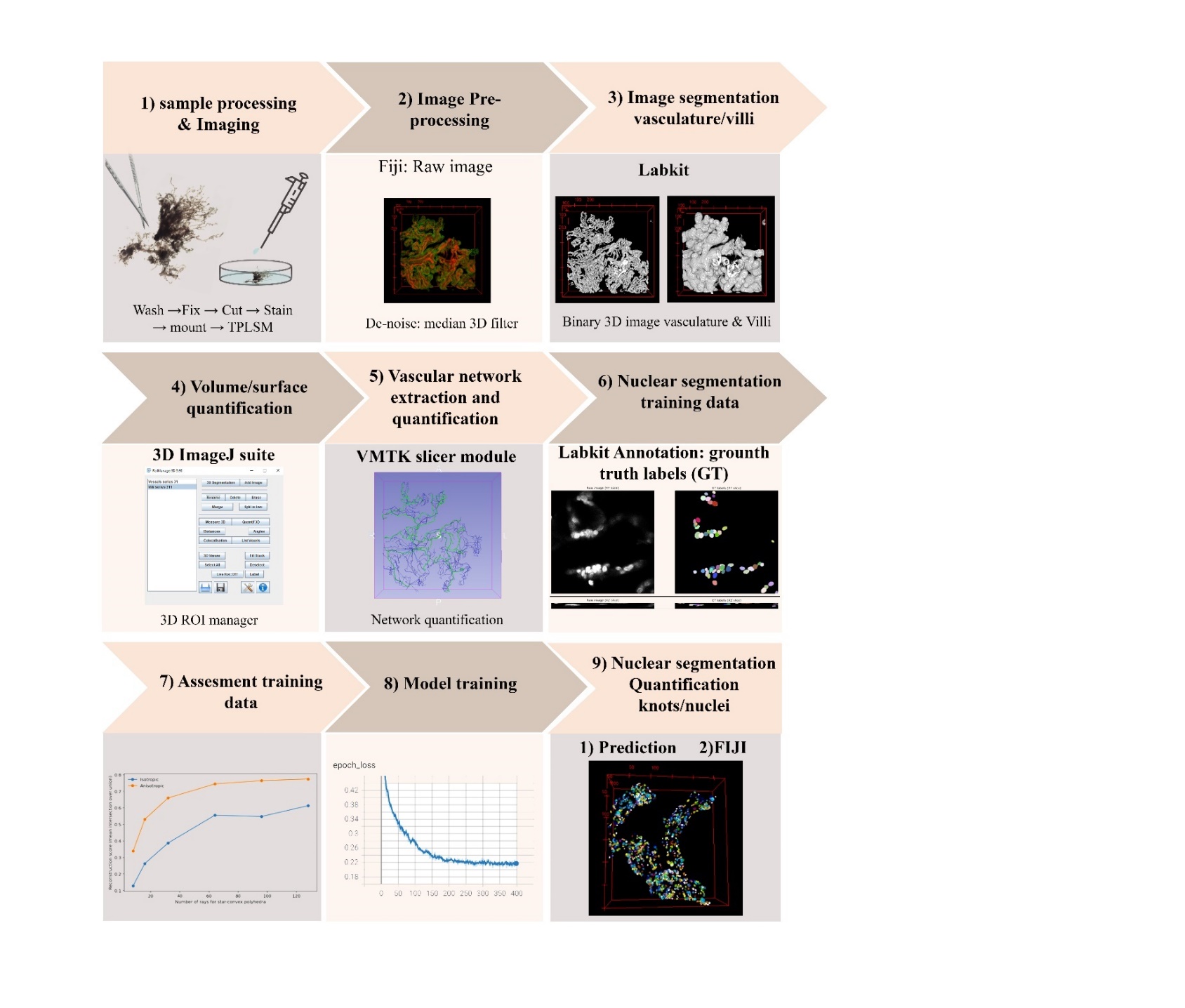


**Figure S4: Imaging, processing and quantification development pipeline for MPM of placental tissue.** Following image acquisition (step 1) the image was pre-processed by denoising in Fiji (step 2). Vasculature and villi were then segmented with the Fiji plugin Labkit (step 3). Volumetric- and surface measurements were performed with the 3D ImageJ suite plugin (step 4). Vascular networks were extracted with VMTK and extracted networks were automatically quantified (step 5). Nuclear segmentation was performed by training a neuronal network with manually annotated image stacks (step 6-8). Lastly, this model was used to predict nuclear locations the images, from which densely clustered nuclei could be selected by KnotMiner (step 9).

**
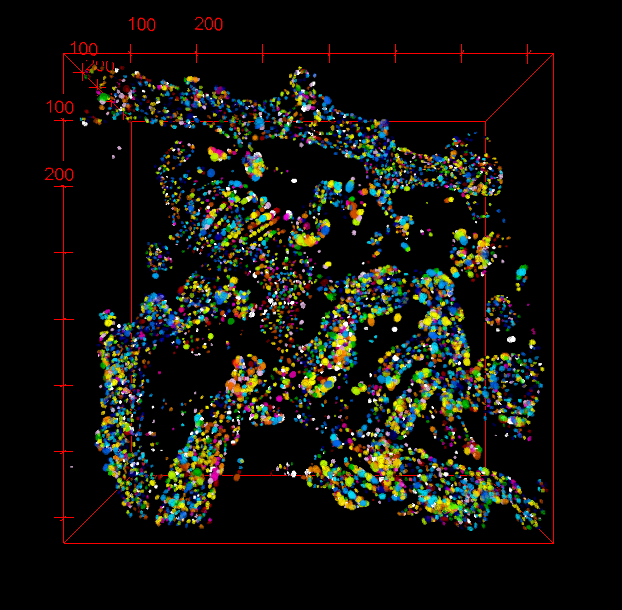

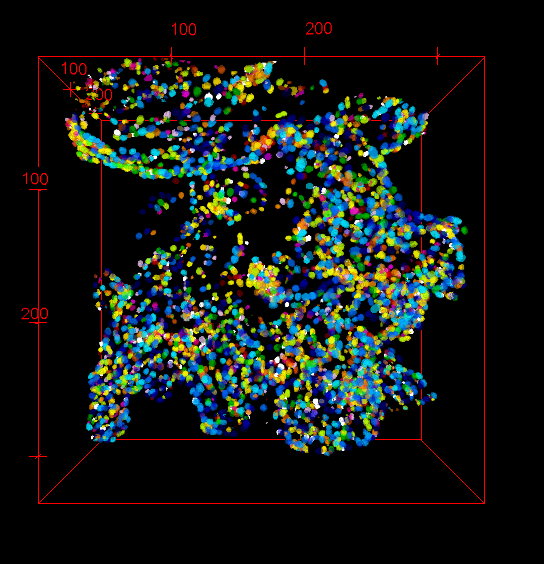

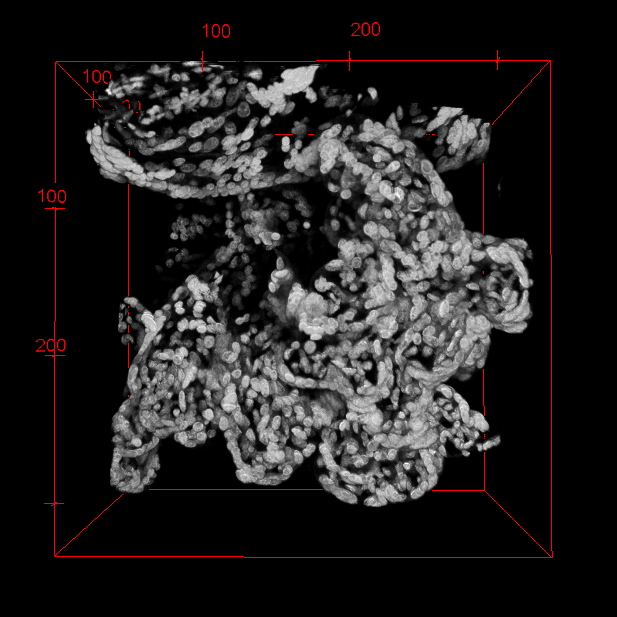

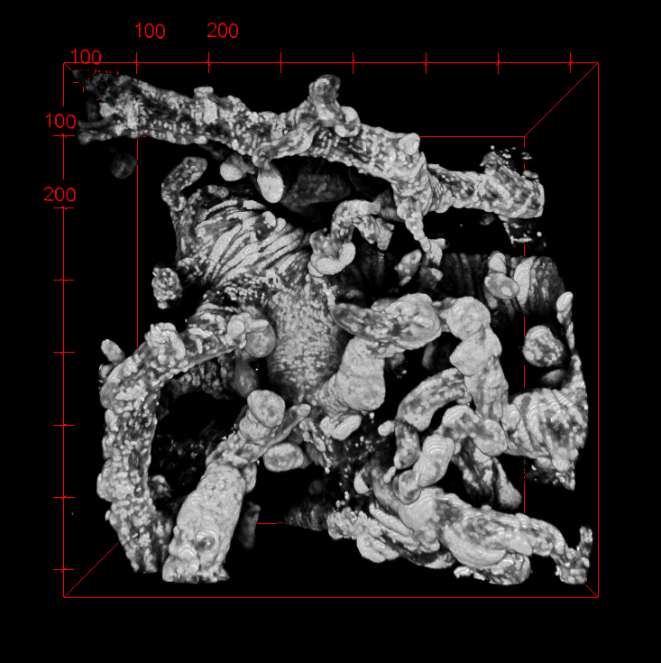
**

**Figure S5**: 3D representation of nuclear segmentation by Stardist, enlarged.

Term control (40w,4d)

RAW

Nuclei segmentation

EO-PE (31w,1d)


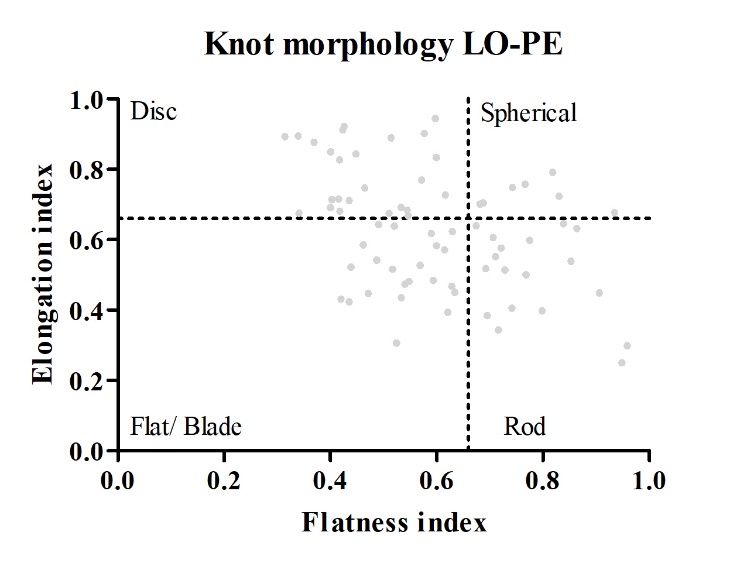

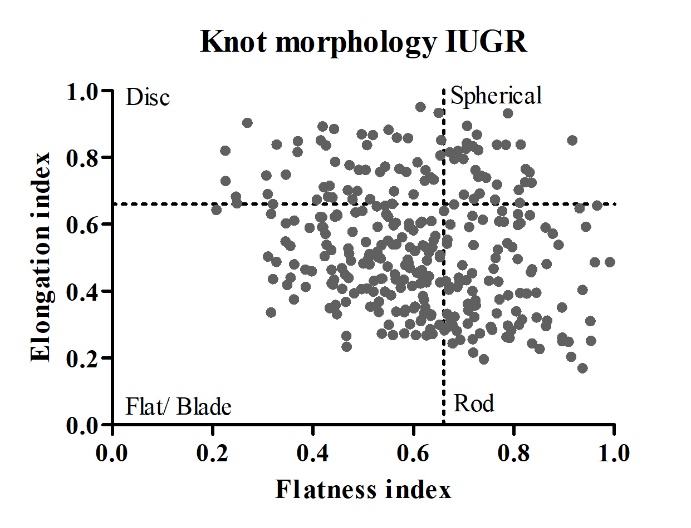

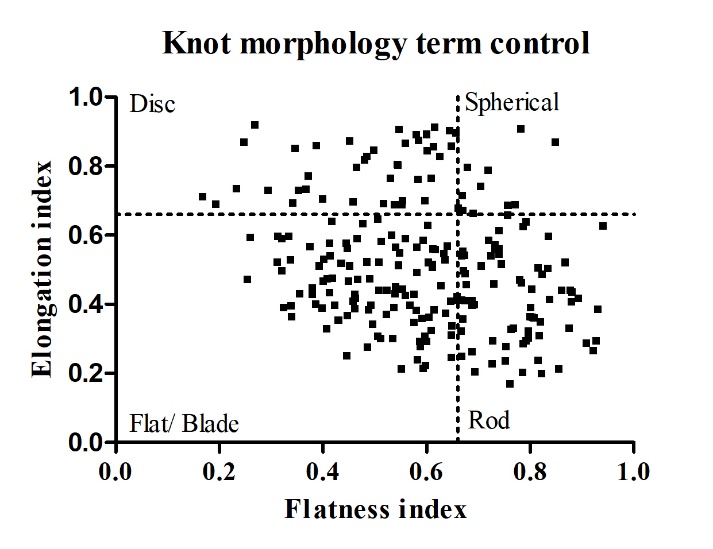

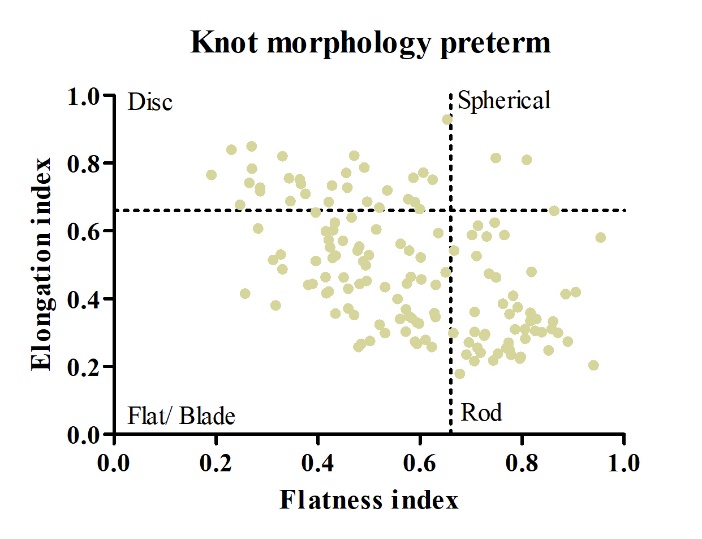

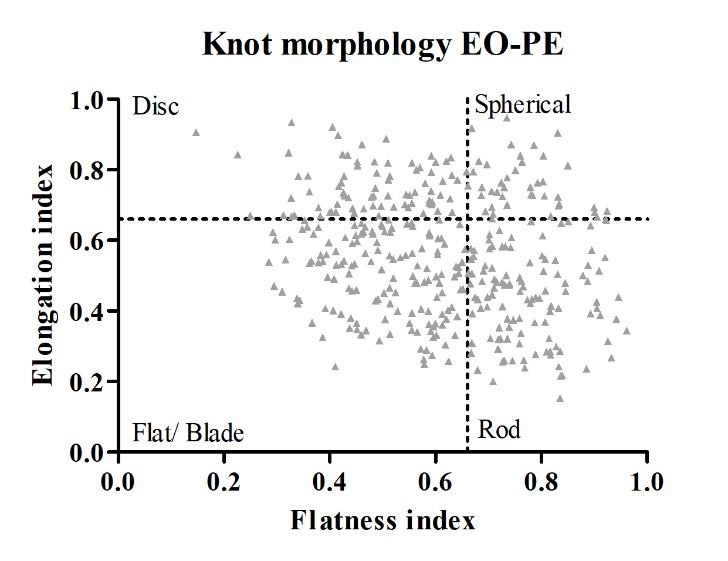


**Figure S6: Morphological analysis of individual knots.** Sprouts and bridges were excluded from this analysis. Nonetheless, their shape could be assessed similarly in the future. Elongation index (EI) of each syncytial knot was calculated by dividing its long principal axis over its intermediate principal axis. The flatness index was calculated by dividing the short principal axis and the intermediate principal axis. Shape subdivision was made according to Zingg (7).

**EO-PE EO-PE EO-PE LO-PE**


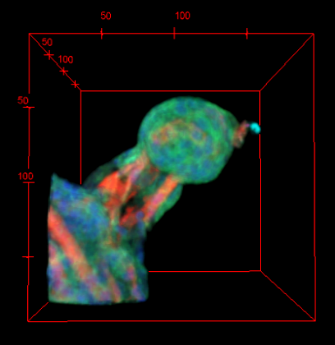

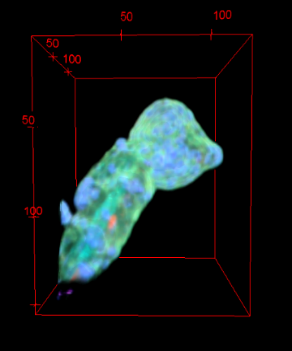

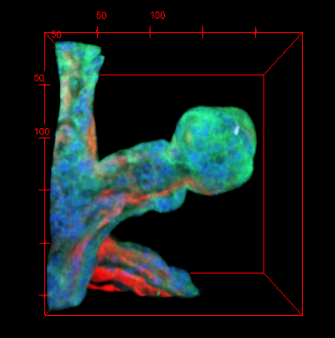

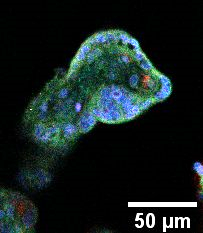

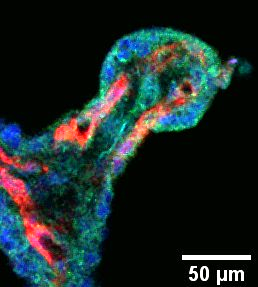

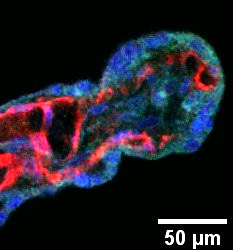

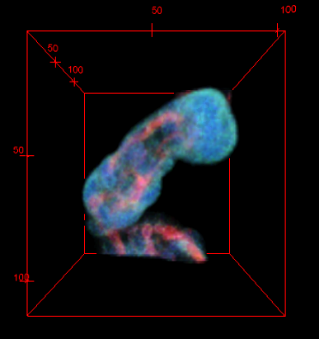

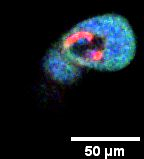


**LO-PE:**

3D

2D

**
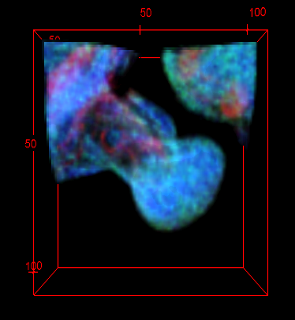

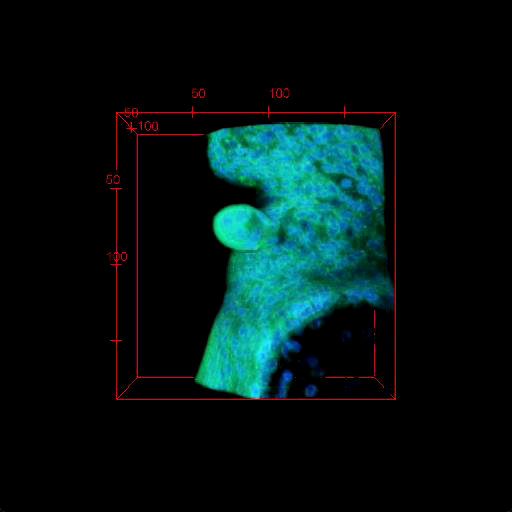

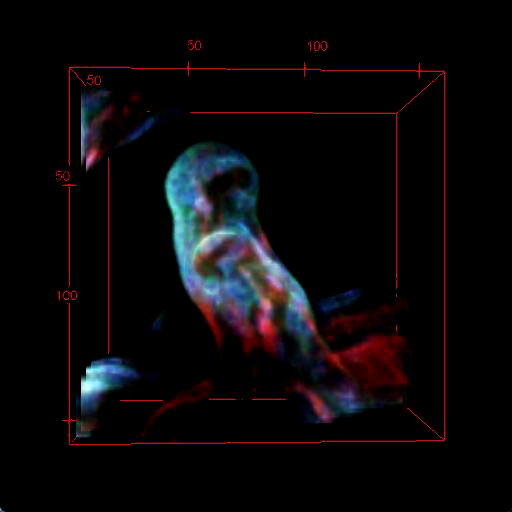
**
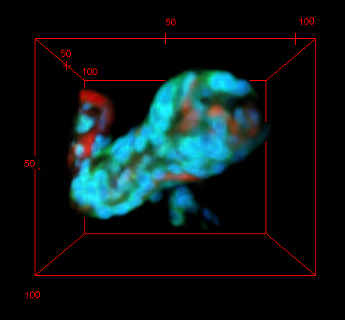
 **Term control Preterm (31w) Preterm (32w) 2^nd^ trimester (22w)**

3D


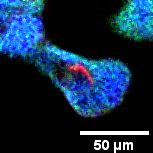
**
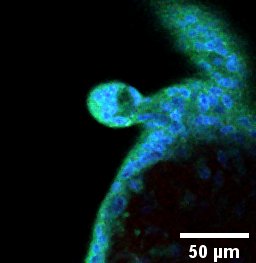

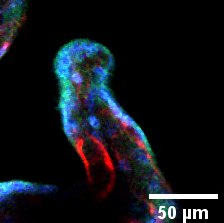
**
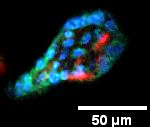


2D

**
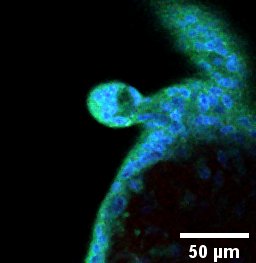
**

**Figure S7: Paddle-like villous endings/ alternate sprout shapes in EO-PE and LO-PE placenta.** Paddle-like villous terminals in EO (30w,1d) and LO-PE (37w,5d) placenta. The shapes were especially obvious and large in EO-PE. Continuation of vasculature suggests them to be sprout like structures. Although less prominently present, less bulbously shaped and smaller, a similar sprout structure was observed in term control placenta (40w,4d), preterm control placenta (31w,5d and 32w,5d) and 2^nd^ trimester placenta (22w,6d). Scale bars: 50µm.


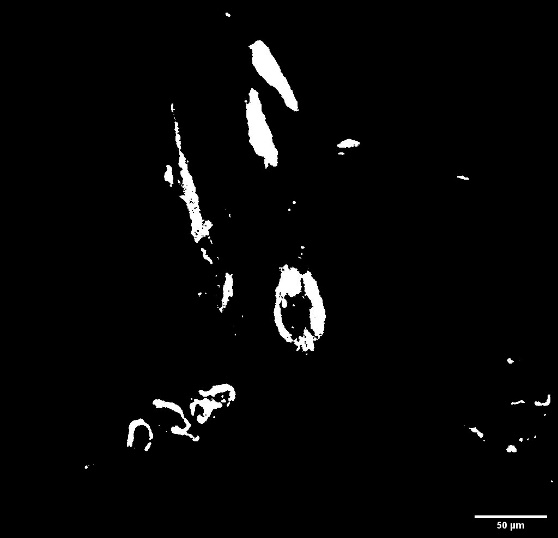

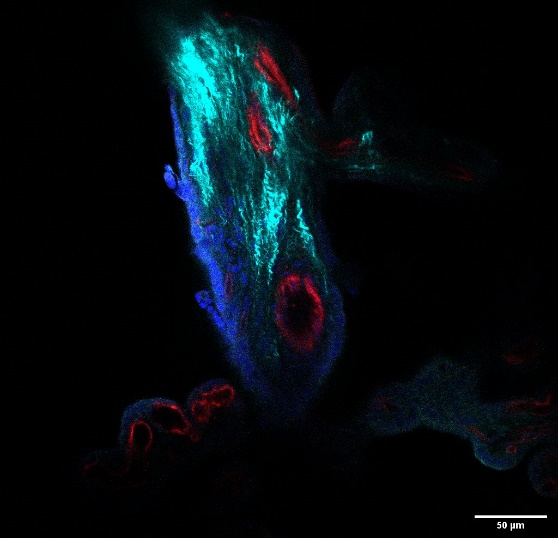

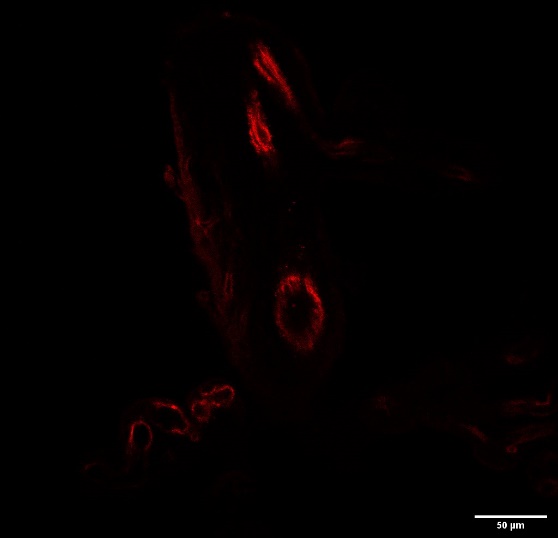

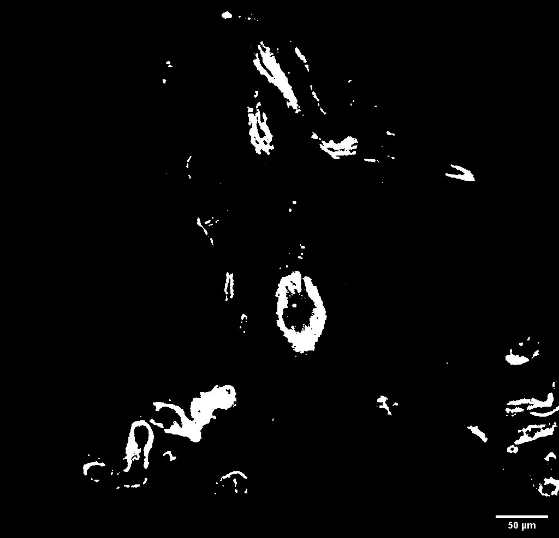


(B)

(A)

*****

(C)

(D)

**Figure S8: Segmentation of vasculature with Labkit.** A: composite of the original 4 channel image, B: vascular staining channel. C: Segmentation of the vascular channel only. the arrow indicates over segmentation because of non-specific membrane staining. D: segmentation of vasculature from the multichannel image. The asterisk shows that large vessel lumens are segmented as background. Vascular segmentation works best when segmenting autofluorescence and vessel channels simultaneously, to prevent slight non-specific membrane staining ((C), arrow) from being segmented as vasculature. Scale bars: 50µm.


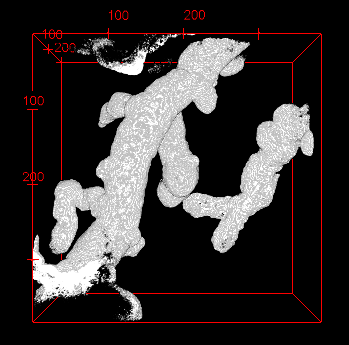

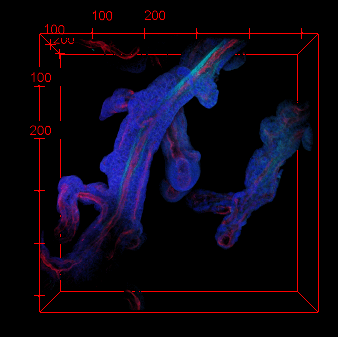

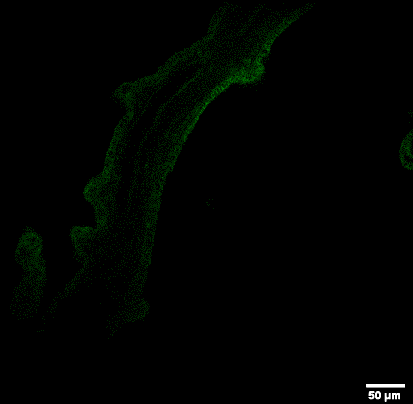

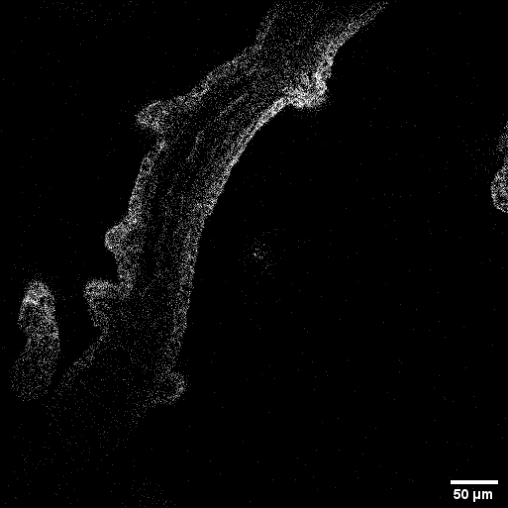

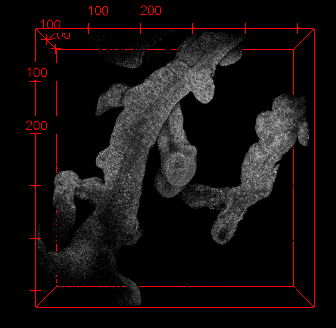


(A)

(B)

(C)

(D)

(F)


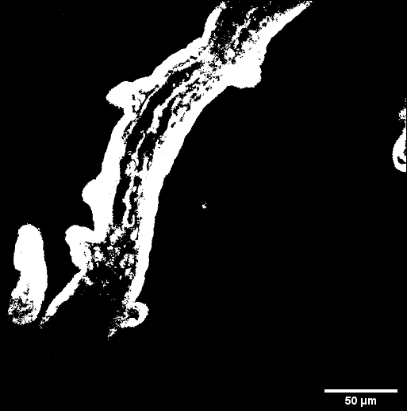


(E)

**Figure S9: Comparison of simple thresholding and Labkit segmentation in images with low SNR autofluorescence signal.** A: 2D slice from a stack made from EO-PE placenta (30w,1d), with low SNR for the autofluorescence channel. Simple thresholding yields non-sufficient results, i.e., too little pixels representing villous tissue. Brightness of the image was increased until almost maximum before segmentation. B: thresholded image, C: 3D representation of the thresholded image. D: shows the 3D representation of the original 4 channelled image, in which autofluorescence signal is clearly overpowered by blue nuclear signal. In E the segmented result with Labkit is shown in a single slice and in F the 3D representation. Scale bars: 50µm.

(D)

(C)

(B)

(A)


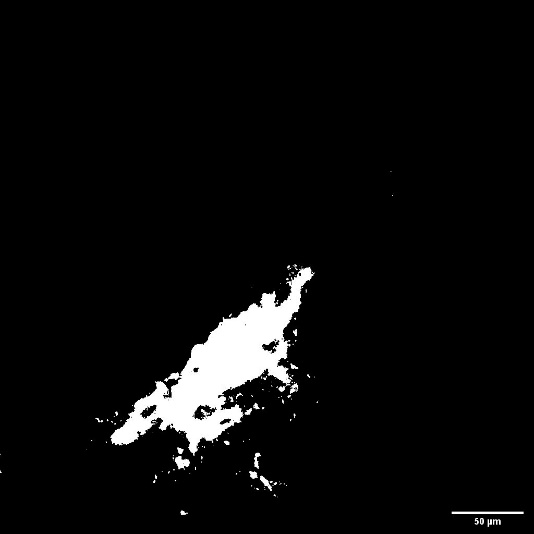


Multichannel image imported for SHG segmentation


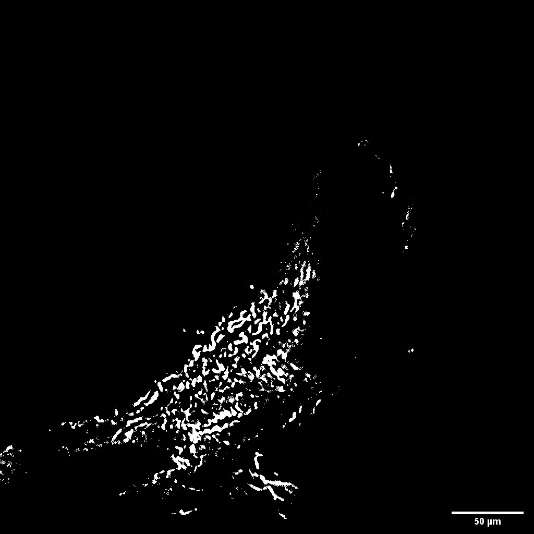


SHG channel imported for SHG segmentation


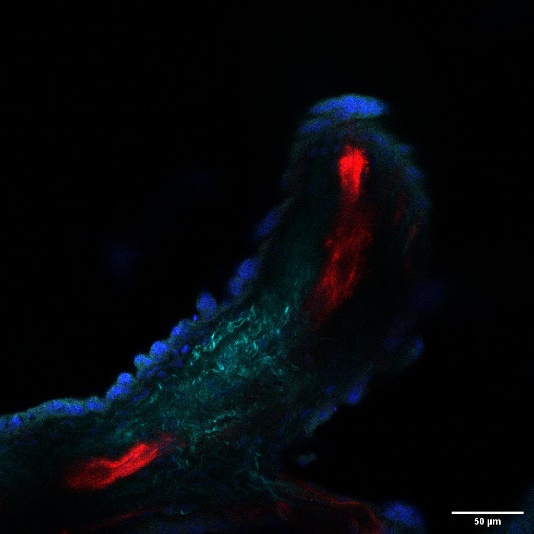


Original multichannel image


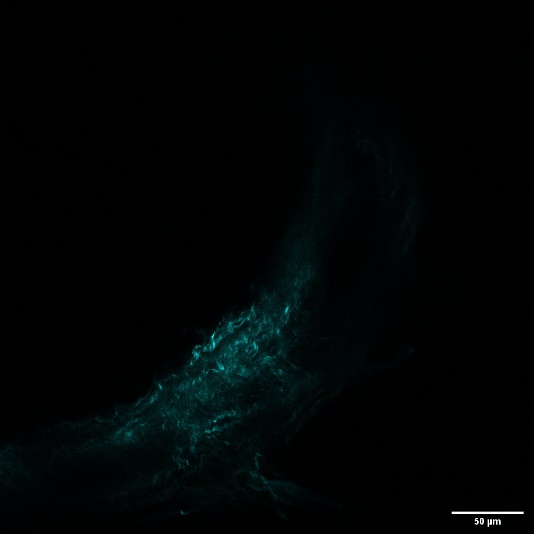


SHG channel only


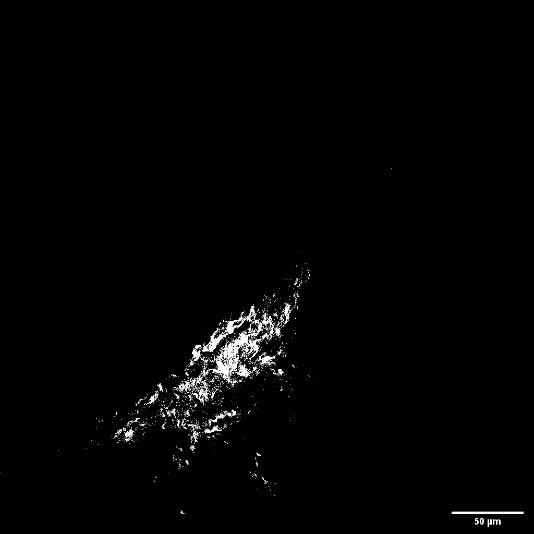


Simple thresholding SHG channel

**Figure S10: Segmentation of collagen (Second harmonic generation) by Labkit.** SHG signal segmentation works best when only loading the SHG channel into Labkit. Labkit segmentation result is superior to simple thresholding. Image: P1 pre-eclamptic placenta. Blue: nuclear staining, green: autofluorescence, red: vascular staining, cyan: SHG. Scale bars: 50µm.


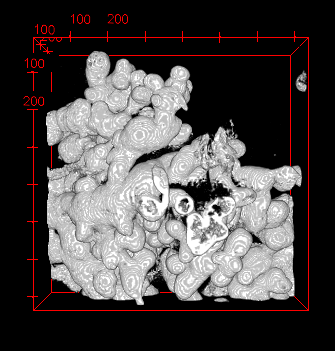

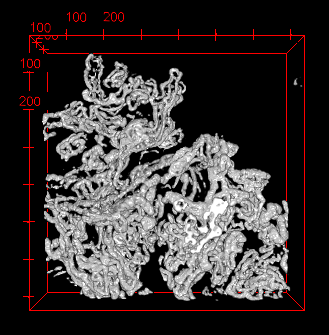

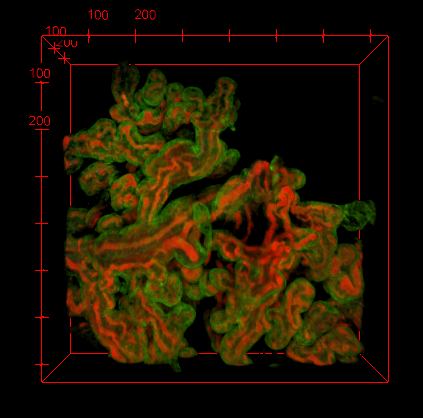


(A)

(B)

(C)

**Figure S11*: Segmentation of villous tissue and vasculature with Labkit.*** Composite image stacks were imported to Labkit to segment villous tissue (autofluorescence signal) and vasculature. The original 3D view of the composite image stack (A) and the 3D segmentation result of vasculature (B) and villous tissue (C) are shown. Green: villous tissue, red: UEA-1 vascular staining.

(G)

(C)

(B)

(A)


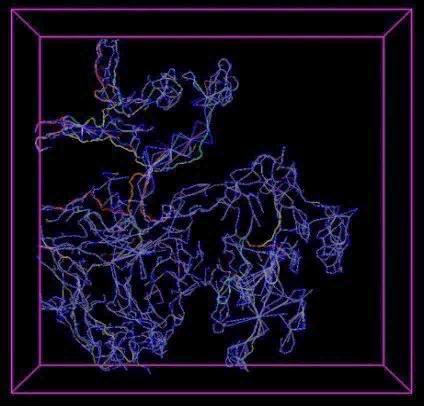

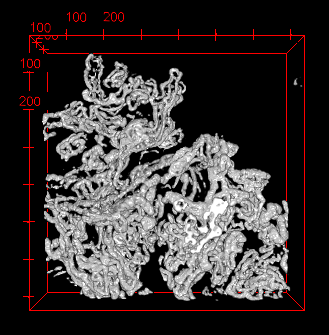

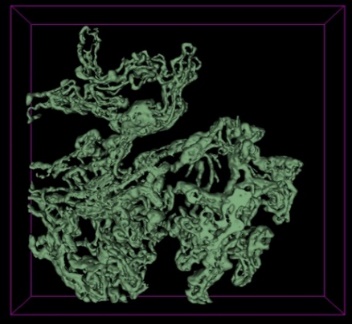

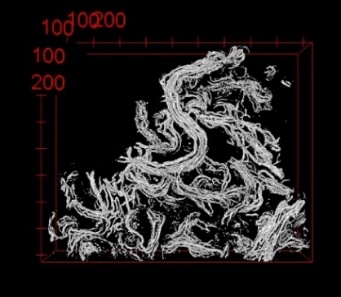

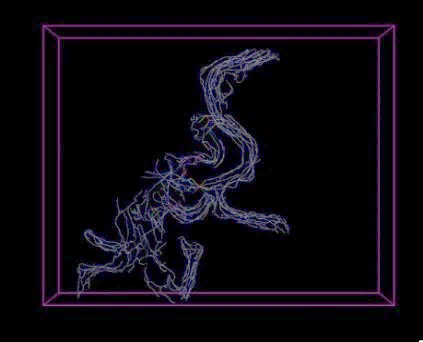

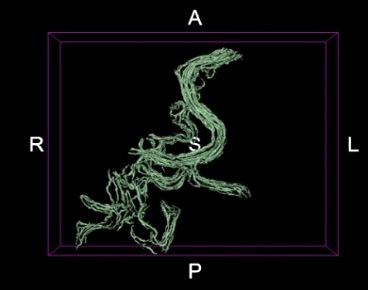


(A)

(B)

(F)

(E)

(D)

(H)

*****

*****


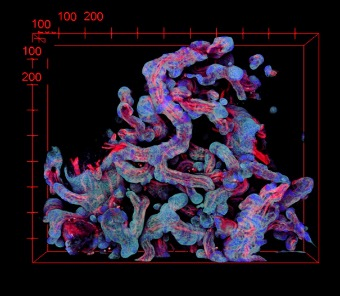

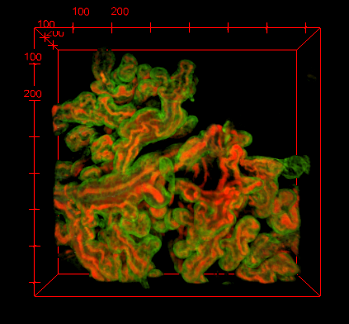


**Figure S12*: Process of 3D network extraction in chorionic villous vasculatur*e.** (A) RAW image of term control placenta (40w,4d). (B) RAW image of LO-PE placenta (37w,5d). Vascular segmentation and network extraction are demonstrated. The binary image (C, D) is imported into the segment editor as .nrrd file, where the largest connected segment is extracted (E, F). A network is then automatically extracted (G, H) and quantitative output for each segment is generated. Asterisks represents possible network extraction mistakes in crowded, overlapping, vascular regions.


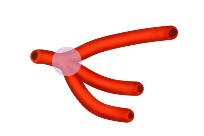
**
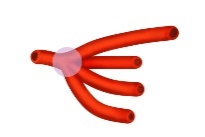
**
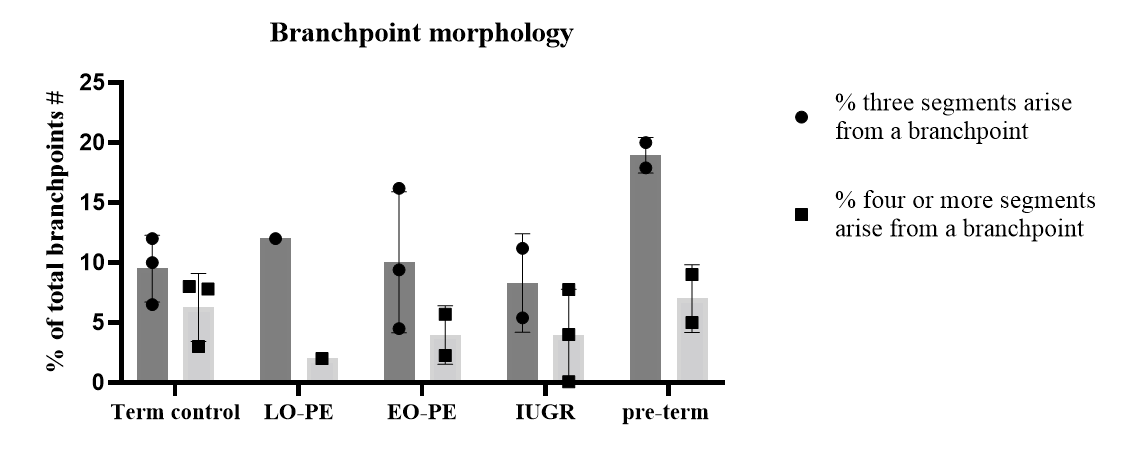

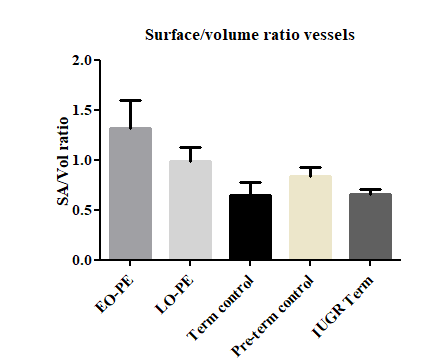
**Figure S13*:*** **Additional parameters that can be extracted by network quantification.** A: Average vessel diameter of a quantified network. B: Average vessel segment length of a total network. In cases of increased branching, shorter segment length would be expected compared to increased non-branching angiogenesis. C: Vessel segment partitioning was calculated by dividing the number of segments and the total network length. In cases of increased branching angiogenesis partitioning is expected to be higher compared to control. The opposite is true for increased non-branching angiogenesis. A-C: n=3 images of EO-PE (30w,1d), n=4 images of LO-PE (37w,5d), n=2 images pre-term control (32w,5d), n=3 images term control (40w,4d) and n=3 images for term, IUGR (40w,0d). D: The surface to volume ratio of vasculature. EO-PE n=7 images (30w,1d), LO-PE n=5 images (37w,5d), term control n=11 images, pre-term control n=4 (32w,1d) IUGR: n=3 images (40w,0d). E: Based on network output, it is also possible to assess the morphology of branchpoints. 2 vessel segments with the same branchpoint coordinate, originate from one branchpoint. Sometimes 3 (triple branchpoint) or 4 (quadruple branchpoints) vessel segments arise in the same branchpoint. This is an additional feature that could be investigated in the future. F: Vessel diameter of LO-PE image presented in main article Figure 4A and G: Vessel diameter of term control image presented in main article Figure 4B. ANOVA, p<0.05*. Error bars: SEM.


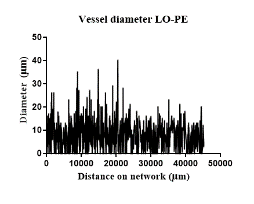

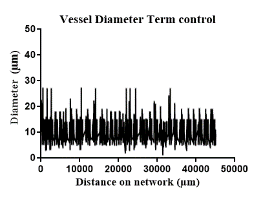


Vessel diameter - Term control

Distance on network (μm)

Diameter (μm)

Diameter (μm)

Distance on network (μm)

Vessel diameter - LO-PE

F

G


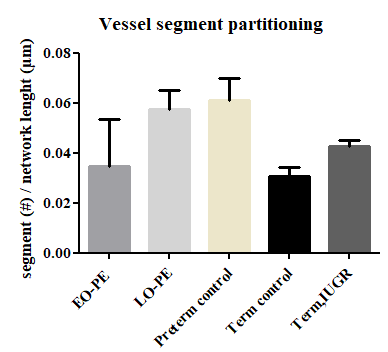

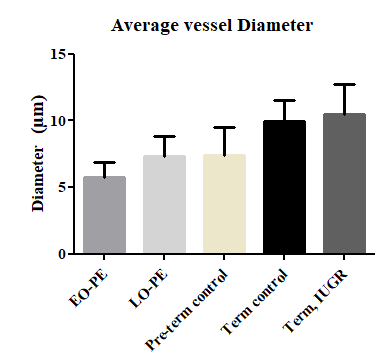

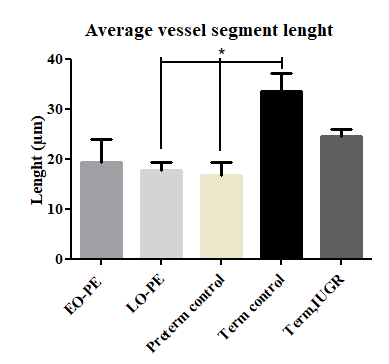


D

E

C

B

A


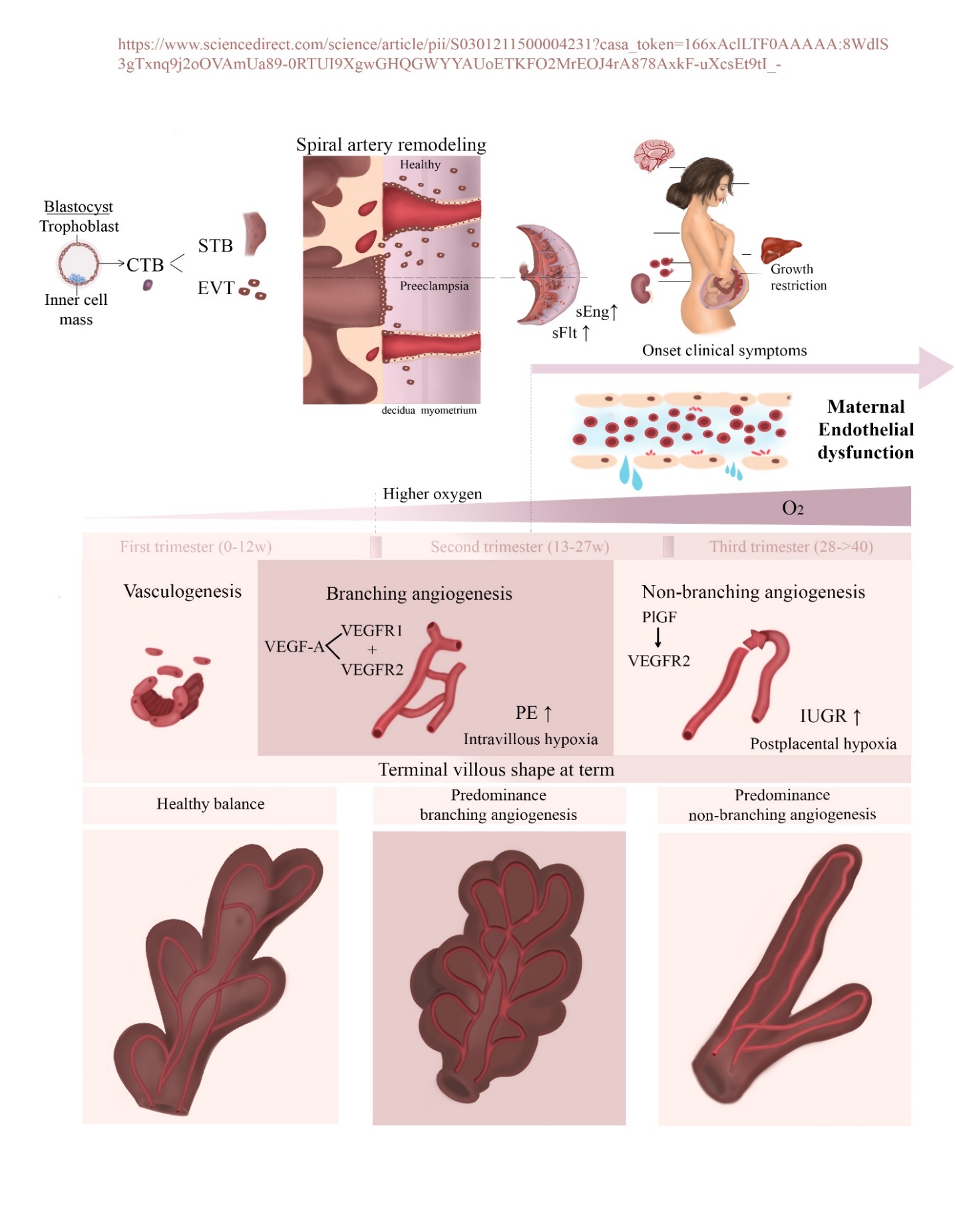


EO-PE/

LO-

**Figure S14: Overview of current hypothesis regarding vascular development in early onset- and late onset pre-eclampsia.** Villous capillaries experience different developmental phases throughout gestation. After vasculogenesis, branching angiogenesis predominates in the first half of pregnancy. During the last trimester of pregnancy non-branching angiogenesis dominates (8, 9). Angiogenetic processes are hypothesised to influence the shape of terminal villi. Oxygen (and subsequent signalling molecule expression) is central for regulating the type and degree of fetoplacental angiogenesis. Disbalanced coordination of vascular patterning will influence the shape and number of terminal villi, which can influence placental efficiency/ diffusion surface area.

**Video S1 :** 3D view of a section of a LO-PE placenta with highly varying regions, with both limited branching and highly branched villi. Image size is 2 X 2.5 X 0.3 mm^3^ (Video uploaded separately)

**Supplementary References**

1. Hypertension in pregnancy. Report of the American College of Obstetricians and Gynecologists’ Task Force on Hypertension in Pregnancy. Obstet Gynecol. 2013;122(5):1122-31.

2. Ives CW, Sinkey R, Rajapreyar I, Tita AT, Oparil S. Preeclampsia—Pathophysiology and Clinical Presentations: JACC State-of-the-Art Review. Journal of the American College of Cardiology. 2020;76(14):1690-702.

3. Ester M, Kriegel H-P, Sander J, Xu X, editors. A density-based algorithm for discovering clusters in large spatial databases with noise. kdd; 1996:226-231

4. Vangrieken P, Vanterpool SF, van Schooten FJ, Al-Nasiry S, Andriessen P, Degreef E, et al. Histological villous maturation in placentas of complicated pregnancies. Histology and histopathology. 2020;35(8):849-62.

5. Orabona R, Donzelli C, Falchetti M, Santoro A, Valcamonico A, Frusca T. Placental histological patterns and uterine artery Doppler velocimetry in pregnancies complicated by early or late pre‐eclampsia. Ultrasound in Obstetrics & Gynecology. 2016;47(5):580-5.

6. Sankar KD, Bhanu PS, Kiran S, Ramakrishna B, Shanthi V. Vasculosyncytial membrane in relation to syncytial knots complicates the placenta in preeclampsia: a histomorphometrical study. Anatomy & cell biology. 2012;45(2):86-91.

7. Zingg T. Beitrag zur schotteranalyse: ETH Zurich; 1935.

8. Ahmed A, Dunk C, Ahmad S, Khaliq A. Regulation of placental vascular endothelial growth factor (VEGF) and placenta growth factor (PIGF) and soluble Flt-1 by oxygen--a review. Placenta. 2000;21 Suppl A:S16-24.

9. Kaufmann P, Mayhew TM, Charnock-Jones DS. Aspects of Human Fetoplacental Vasculogenesis and Angiogenesis. II. Changes During Normal Pregnancy. Placenta. 2004;25(2):114-26.
